# Implicit Theories about Athletic Ability Modulate the Effects of If-Then Planning on Performance in a Standardized Endurance Task

**DOI:** 10.3390/ijerph17072576

**Published:** 2020-04-09

**Authors:** Anna Hirsch, Maik Bieleke, Julia Schüler, Wanja Wolff

**Affiliations:** 1 Sport Psychology, Department of Sport Science, University of Konstanz, 78464 Konstanz, Germany; julia.schueler@uni-konstanz.de (J.S.); wanja.wolff@uni-konstanz.de (W.W.); 2 Educational Psychology, Department of Psychology, University of Vienna, 1010 Vienna, Austria; maik.bieleke@univie.ac.at; 3 Educational Psychology, Institute of Educational Science, University of Bern, 3012 Bern, Switzerland

**Keywords:** muscular endurance performance, self-regulation, implementation intentions, psychobiological model, Borg scales, implicit theories, limits of athletic performance

## Abstract

Muscular strength has a strong positive impact on cardiometabolic health and fitness. However, building up strength endurance requires effortful exercises. From a health perspective, it is important to understand which psychological strategies help people deal with straining exercise. Self-regulation strategies like if-then planning (also known as implementation intentions) appear particularly promising because they might directly alter how people deal with exercise-induced sensations. However, research on the effects of if-then planning on exercise performance has yielded mixed results so far. One possible reason for these inconsistent results is the lack of tailored interventions and the neglect of potential moderators. To address this, we investigated the efficacy of if-then plans that were tailored to perceived limits of endurance performance (i.e., perceptions of exertion versus pain). In addition, we investigated the effects of these tailored if-then plans while taking into account the potentially moderating effects of individual differences in implicit theories. Specifically, we were interested in the role of implicit theories about athletic performance (i.e., entity versus incremental beliefs) and about the limitation of athletic performance by mental versus physical factors (i.e., mind-over-body beliefs). *N* = 66 male students (age: *M* = 25.8 years, *SD* = 3.2) performed a static muscular endurance task twice (measurement: baseline task vs. main task) and were randomly assigned to a goal or an implementation intention condition. They were instructed to hold two intertwined rings for as long as possible while avoiding contacts between them (measure of performance: time-to-failure and errors). After the baseline task, participants were either given an implementation intention or were simply asked to rehearse the task instructions. The content of the instruction depended on whether they ascribed ultimate baseline task termination to perceptions of exertion or pain. After the main task, implicit theories on athletic ability were assessed. No differences in performance emerged between conditions. In the implementation intention condition, however, stronger entity beliefs were associated with increasing time-to-failure when participants planned to ignore exertion but with decreasing time-to-failure when they planned to ignore pain. This pattern of results was reversed with regard to mind-over-body beliefs. These findings indicate that the efficacy of psychological strategies hinges on recreational athletes’ beliefs regarding athletic performance.

## 1. Introduction

Research shows that physical fitness contributes strongly to the current and future health of adults and children alike [1,2]. Even though most work has focused on the importance of cardiorespiratory fitness for health outcomes, muscular fitness is increasingly recognized as a relevant factor as well [3]: it is linked to lower cancer mortality risk in men [4], lower overall mortality [5], as well as enhanced bone health, boosted self-esteem, and reduced adiposity in children [3,6]. Although these benefits are widely known and despite recommendations by major health organizations to engage in muscular endurance training [7] to enhance cardiometabolic health and fitness [8], a large number of people is still not active: an estimated 77% of U.S. citizens do not fulfill physical activity recommendations on cardiovascular and strength training [9]. To complicate matters further, building up muscular strength requires to endure muscular exercises for a sufficient time at an optimal intensity, which might be perceived as too effortful and encourage premature termination of an ongoing exercise. From a health perspective, it is therefore paramount to understand which factors influence and eventually limit muscular endurance performance and whether there are strategies to effectively deal with these factors.

A large body of research investigated physiological limits to exercise tolerance [10,11,12,13] and suggests that people might not fully deplete their physiological resources in straining exercise [14,15]. This points toward an important role of psychological factors in exercise termination. For example, according to the psychobiological model of exercise tolerance [16,17], perceived exertion critically limits endurance performance. The model states that endurance exercises are terminated when applying additional effort seems either unjustified or impossible [16,17]. Applied to muscular endurance performance, this suggests increasing self-regulatory demands [18]: as upholding certain levels of performance becomes more and more difficult (i.e., the perception of exertion rises), self-regulatory challenges rise as well (e.g., enduring increasing sensations of pain).

One strategy that might help to deal with these challenges is the self-regulatory strategy of if-then planning (also referred to as forming implementation intentions [19,20]), which has already been investigated in the context of health (i.e., [21,22,23]) and various other domains [24]. In an if-then plan, a person specifies how to achieve a goal (e.g., enduring an effortful task). It comprises selecting a goal-relevant situation (e.g., upcoming sensations of muscle pain) and linking it to a goal-directed behavior (e.g., ignoring the pain and continuing the exercise) in an if-then format: “If I encounter Situation S, then I will perform Behavior B!” (e.g., “And if my pain becomes too great, then I tell myself: I can still keep going!”). Making if-then plans is assumed to activate the mental representation of the situation which thereby becomes easier to remember and to recognize [25,26]. Research further indicates that if-then planning automates the initiation of the goal-directed behavior [19,20]. Accordingly, implementation intentions are seen as an efficient bottom-up form of action control compared to goal intentions, which merely specify what to do but not how to do it (e.g., “I want to ignore the pain and continue the exercise!”) [27] and are thus less conducive to automatic behavior initiation. This is an essential aspect especially in complex health behaviors like exercising that confront people with various barriers to perform successfully and to keep exercising.

In support of this notion, one study showed that implementation intentions help athletes to recognize opportunities for hydration and to increase the intake of carbohydrate-electrolyte solutions during a cycling exercise by over 50% [28]. In a study with tennis players, implementation intentions on how to deal with intrusive thoughts, emotions and physiological states led to improved performance during matches [29]. More relevant for our present purposes, however, are investigations of the effects of if-then planning in muscular endurance tasks. This research is scarce, though, and has so far produced inconsistent results. On the one hand, if-then plans helped participants deal with perceptions of pain in a ball-holding task: Planning to direct attention away from muscle pain and to initiate self-affirmative speech (“And if my muscles hurt, then I will ignore the pain and tell myself: I can do it!”) led to an increase in time-to-failure compared to a condition with a corresponding goal intention [30]. On the other hand, two similar studies focusing on time-to-failure in a rod-holding task found no effects of if-then planning on performance. In one study [31], planning to ignore perceived exertion did not alter performance and actually increased exertion. Such a null-finding also emerged in the second study [18] in which participants planned to ignore perceptions of pain (as in the ball-holding study)—although functional near-infrared spectroscopy (fNIRS) during task performance showed lower lateral prefrontal cortex activity among participants in the implementation intention condition, indicating lower activation in an area that is critical for effortful self-regulatory control. Taken together, these results indicate that if-then planning affects the correlates of muscular endurance performance but not necessarily performance itself. Furthermore, it suggests that neither targeting perceptions of exertion nor targeting perceptions of pain yields consistent effects of implementation intentions.

This poses the question of what might cause the inconsistent findings. It seems worthwhile to investigate whether the effects of if-then planning are moderated by factors that were not yet accounted for. Here, we focus on two of these factors: First, it might matter whether people experience perceptions of exertion or perceptions of pain as limits to their own performance. Exertion refers to “the conscious sensation of how hard, heavy, and strenuous a physical task is” [32] and therefore reflects mental processes (e.g., the urge to quit). Pain, on the other hand, is defined as “an unpleasant sensory and emotional experience associated with actual or potential tissue damage.” [33] and thus reflects bodily processes (e.g., locomotor muscle fatigue or accumulation of lactic acid). Previous research provided the same plan to all participants (i.e., dealing with pain or dealing with exertion) and therefore does not provide information on whether and how individual differences in experiencing mental versus bodily limits obscure implementation intention effects.

Second, it might matter to which degree participants believe that performance is generally limited versus improvable, and whether potential limits pertain to the mind versus the body. Such beliefs are commonly referred to as implicit theories that people may hold regarding attributes like intelligence [34]. These beliefs are commonly divided into two categories that describe how people judge the malleability of an attribute [35,36,37]: People are said to hold entity beliefs if they think that an attribute is fixed and stable, while they are said to hold incremental beliefs if they think that an attribute is flexible and changeable. These beliefs influence decisions, for example regarding what goals to pursue [36], but are usually unconscious [38]. People holding entity beliefs seek positive appraisals of their expertise and thus favor performance-oriented goals, while people holding incremental believes try to enhance their ability through acquirement and thus favor learning-oriented goals [35]. Learning-oriented goals are often (but not necessarily) associated with more beneficial outcomes than performance-oriented goals because they are conducive to using negative feedback as a means for further development rather than interpreting it as a mere sign of lack of ability. Considering research on implicit theories about willpower, it is implied that failing self-regulation after a short execution of self-control does not originate in people’s actual self-control resources but is defined by their beliefs on how much self-control they are able to exert [39]. This research underlines the important role which implicit theories have on behavior, as they for example disclose failures in self-control [40].

This theorizing can be transferred to the exercise context, in which implicit theories pertain to the malleability of athletic abilities and thus influence exercise behavior [41,42]. Specifically, people holding incremental beliefs see athletic ability as changeable by learning, effort, and training, whereas people holding entity beliefs see it as innate and stable, and thus unchangeable by training and effort [42,43]. Interestingly, a person can hold both incremental and entity beliefs at the same time, thinking of athletic ability as an inherited capability that can still be enhanced through commitment and effort [43]. Incremental beliefs about athletic ability are connected to higher self-driven motivation, enjoyment and grit as they are accompanied by expectations of improved performance [42,44]. Entity beliefs, on the other hand, are more likely to lead to frustration and discouragement because they render failure as feedback on a lack of ability [42,44].

It is therefore plausible that entity and incremental beliefs moderate implementation intention effects in various ways. For instance, entity theorists could undermine their effects due to doubts that planning improves a performance that one deems unchangeable. Alternatively, people holding entity beliefs might be more likely to respond well to an external strategy (i.e., if-then plans) as means to avoid failure in the upcoming performance (and thus avoid feedback on a lack of ability [45]). People with incremental theories might, in contrast, be amenable to if-then plans because they consider changes in performance as generally possible and might be more receptive to learning for improvement [46]. They may, however, perform better regardless of external self-regulatory strategies, as they are shown to be more persistent in challenging exercise tasks compared to entity theorists [47]. Given these considerations, it is plausible that implicit theories affect implementation intention efficacy. To our knowledge, this interaction has not been addressed so far.

Moreover, the interaction between beliefs and implementation intentions could be even more intricate when we consider that participants might perceive exertion or pain as limit to their performance. As we have argued above, exertion and pain serve as signals that mental or bodily limits have been reached, respectively. To understand the (lack of) implementation intention effects, it might therefore be important to know whether people believe that performance is ultimately limited by mental (mind) rather than by physical factors (body)—which we refer to as mind-over-body beliefs. For instance, people who perceive the body as limiting factor in athletic performance (i.e., low mind-over-body beliefs) might interpret pain as a signal that this limit is reached and deem further attempts to improve futile. This might undermine the effectiveness of planning to simply ignore pain as a means to endure longer. This could be different for people who believe that performance is ultimately limited by mental factors (i.e., high mind-over-body beliefs). To them, pain does not signal that a performance limit is reached, rendering plans targeting the regulation of pain more auspicious. Analogous arguments can be constructed with respect to exertion: people holding low (versus high) mind-over-body beliefs might not (versus might) identify perceptions of exertion as a limiting signal, which might increase (versus decrease) the efficacy of planning to ignore exertion. In sum, the effectiveness of plans targeting the regulation of exertion versus pain might depend on people’s beliefs about athletic ability (entity, incremental, mind-over-body), their perception of limiting sensations during an endurance task (pain, exertion), and the interaction of beliefs and limits.

Taken together, the aim of this study was twofold. First, we wanted to improve upon previous research designs. In previous studies, all participants received the same if-then plan that targeted either perceived exertion [31] or pain [18,30]. These studies did not take into account whether these sensations were in fact limiting the performance of individual participants. To address this shortcoming, we asked participants to state whether they had terminated the baseline task because of exertion or pain and then gave them an if-then plan that was tailored to their individual reason for task termination. Second, we wanted to investigate whether the efficacy of if-then planning is influenced by participants’ implicit theories about athletic ability. While there are studies on the influence of entity and incremental beliefs on the motivation in physical activity and enjoyment of sports (e.g., [42,44,48]), we are not aware of studies on the importance of implicit theories for the effectiveness of self-regulation strategies. As we have outlined above, there are several ways in which the beliefs resulting from implicit theories might interact with plan effects and with sensations of exertion or pain. For instance, entity and incremental beliefs could be differentially conducive to planning effects. Moreover, beliefs about physical versus mental limits of performance could affect implementation intention effects depending on whether people struggle with sensations of pain or exertion.

Beyond these two major aims, studies on if-then planning effects on endurance performance were so far afflicted by a high inter-individual variability especially with regard to time-to-failure (which is common in endurance performance research, see [49,50]). This variability might have obscured potential differences between goal and implementation intention conditions. We therefore added a baseline measure that allowed us to account for preexisting differences in endurance performance. These changes—increased statistical power with a baseline measure and if-then plans tailored to perceived limits of endurance performance (i.e., perceptions of exertion versus pain) in combination with potentially moderating effects of participants’ individual beliefs about athletic ability (entity, incremental, mind-over-body)—should further advance our understanding of when and how if-then planning affects endurance performance.

## 2. Materials and Methods

### 2.1. Participants and Design

We recruited an all-male sample of *N* = 66 participants (age: *M* = 25.8 years, *SD* = 3.2) to minimize gender-related variance in endurance performance [31,50]. This sample size allows us to detect medium-to-large differences (*d* = 0.70; a typical effect size of implementation intentions is *d* = 0.65, see [19]) between the goal and the implementation intention condition in two-sided t-tests (α = 0.05). The study adopted a 2-within (measurement: baseline task vs. main task) × 2-between (condition: goal intention vs. implementation intention) design; participants were randomly and equally assigned to conditions. As third factor, we measured whether participants identified perceived exertion or pain as limiting their performance in the baseline task (limit: exertion versus pain). More participants named exertion (48 in total, including 23 in the implementation intention condition) than pain (18 in total; including 10 in the implementation intention condition) as their limit, with a similar distribution across conditions, χ^2^ (1, *N* = 66) = 0.782, *p* = 0.782. Six participants (five in the implementation intention condition) were excluded from data analysis due to deviations from study protocol (the exclusion of those six participants did not change the pattern of results in terms of significance). The remaining participants reported exercising *M* = 6.7 hours per week (*SD* = 4.7), of which 33.3% were related to strength training. Fifty-seven participants stated to be engaged in a variety of sport activities, having performed their main sport activity for an average of *M* = 7.8 years (*SD* = 6.2), while 3 participants reported to be physically inactive (2 in the goal condition). Participants in the goal and the implementation intention condition did not differ in regard to their weekly training hours, *p* = 0.448, or the duration of performing the main sport, *p* = 0.306. When we advertised the study online, we emphasized that no current or recently healed injuries in shoulders, arms, or the back should be present to be eligible for participation. Moreover, we asked to avoid alcohol and strenuous exercise the day before the experiment and to refrain from consuming caffeine in the two hours before. Most participants complied with these requests with no differences between conditions, *p* > 0.545. Specifically, 16 participants (nine in the implementation intention condition) reported injuries that had happened some time ago (three in the last 6 months, four in the last 7–12 months, nine more than 12 months ago). And while all participants complied with the instruction not to consume caffeine, 18 participants exercised (eight in the implementation intention condition) and 16 consumed alcohol (nine in the implementation intention condition) the day before the experiment. All participants signed an informed consent and were compensated with 5 Euro and course credit. The study protocol and measurements were approved by the Ethics Committee at the University of Konstanz (approval #24/2016).

### 2.2. Static Muscular Endurance Task

**Figure 1:**
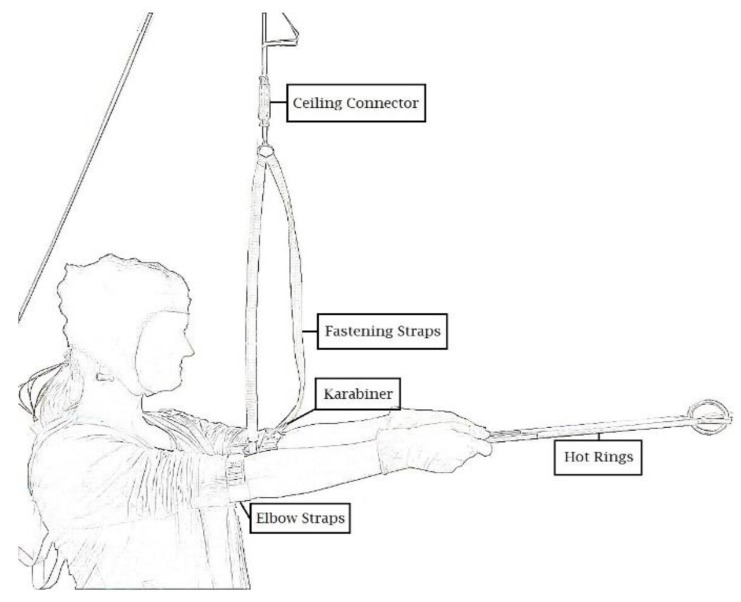
Illustration of the “hot rings task” (HRT; [18,31]).

We used the “hot rings task” (HRT; [31]) to measure static muscular endurance performance. Participants were instructed to hold two aluminum bars connected by intertwined rings for as long (time-to-failure) and with as few contacts between the rings (errors) as possible. As shown in Figure 1, participants stand in an upright position with their arms outstretched to form a 90° angle with their torso. A connector element links participants’ arms with the aluminum rods via a holding device with a recording box for reliably measuring time-to-failure and errors. The recording box measures ring contacts at 50 Hz. The holding device is fastened at the ceiling of the laboratory and can be flexibly adjusted to each participant’s individual height while the connector element is still locked. This way, participants were able to rest their arms during the adjustment period. Before the HRT begins, the connector element is unlocked. It unplugs as soon as participants lower their arms below the preset 90° angle. Time-to-failure is recorded simultaneously with a stopwatch. The HRT allows measuring muscular endurance performance in terms of time-to-failure and errors simultaneously. The total duration of ring contacts (measured by the recording box in milliseconds) was added up to an error score in seconds.

### 2.3. Ratings of Perceived Exertion (RPE) and Pain (RPP)

While performing the HRT, participants were prompted by a recorded computer voice to state RPE and RPP every 25 ± 10 s using Category Ratio 10 (CR10) scales [51,52]. In order to make sure that participants differentiated on what sensation (exertion vs. pain) to focus on [33], RPE was described to them as “the conscious sensation of how hard, heavy, and strenuous a physical task is” [32]. RPP was supposed to be rated according to individual discomfort but was not separately defined to the participants. We printed individual scales for RPE and RPP on sheets of paper, hung on the wall in front of the participants. Each scale ranged from 0 (“nothing at all”) to 10 (“maximal”) or 11 (“even more than max”) [33].

### 2.4. Questionnaires

After both the baseline and the main task, participants stated whether they currently felt several negative (*exhausted, uncomfortable, annoyed, tense;* Cronbach’s α = 0.73) and positive emotions (*happy, energetical, content, relieved;* Cronbach’s α = 0.78) on seven-point Likert scales (*1: does not apply, 7: fully applies*). For the analyses, emotional states were aggregated to positive / negative feelings. Then, they declared their reason for baseline task termination (exertion vs. pain, other reasons). Additionally, they indicated their performance motivation (e.g., “*It was important for me to persist for as long as possible in the endurance task*.”, Cronbach’s α = 0.90), task satisfaction (e.g., “*I am satisfied with how precisely I performed in the endurance task.*”, Cronbach’s α = 0.74), and self-efficacy (e.g., “*I am convinced of my ability to perform endurance tasks like this for as long as possible.”,* Cronbach’s α = 0.86) with respect to both time-to-failure and errors on seven-point Likert scales (*1: does not apply, 7: fully applies*).

After the main task, we measured participants’ implicit theories about athletic ability using the *Conceptions of the Nature of Athletic Ability Questionnaire 2* (CNAAQ-2; [42]). It comprises 12 items (Table 1), with a subscale of six items pertaining to entity beliefs (Cronbach’s α = 0.75) and another subscale of six items pertaining to incremental beliefs (Cronbach’s α = 0.76). We averaged the six items of each subscale into composite scores with higher values indicating a stronger belief that athletic ability is either fixed and stable (entity) or improvable and trainable (incremental), respectively. Following the authors of the questionnaire [42] and considering that incremental and entity beliefs are no orthogonal constructs, we used the two scales separately in the analyses. We generated three additional items (Table 1, Items 13–15) to measure mind-over-body beliefs (Cronbach’s α = 0.55). These three items were averaged into a composite score with higher values indicating that the mind rather than the body is seen as limiting athletic ability. Answers were given on a five-point Likert scale from 1 (*strongly disagree*) to 5 (*strongly agree*). Finally, participants reported a number of demographic information (i.e., age, physical activity, main sports).

### 2.5. Procedure

Each session was carried out by a researcher who first explained the study and the muscular endurance task to participants. Importantly, they were not informed at this point that they would have to perform the task twice to avoid strategic allocations of resources. After adjusting the holding device to participant’s height, the researcher explained the experimental procedure and the CR10 scale. A demonstration trial was provided to give participants a feeling for the sensitivity of the connector element by having them lower their arms below a 90° angle. Then, after participants had stated their initial level of RPE and RPP, the baseline task started. The researcher did not interact with participants during the task and stayed outside their field of vision. Participants were prompted by a recorded voice played by a computer to state their RPE and RPP, while the experimenter documented the answers. As soon as the connector element unplugged, the baseline task ended, and participants stated their final RPE and RPP. They also indicated whether they terminated the baseline task due to exertion or pain. Participants now learned that they would perform the task again after a five-minute resting period. During this time, they answered a questionnaire about their current emotional state, task performance motivation and self-efficacy.

**Figure A1:**
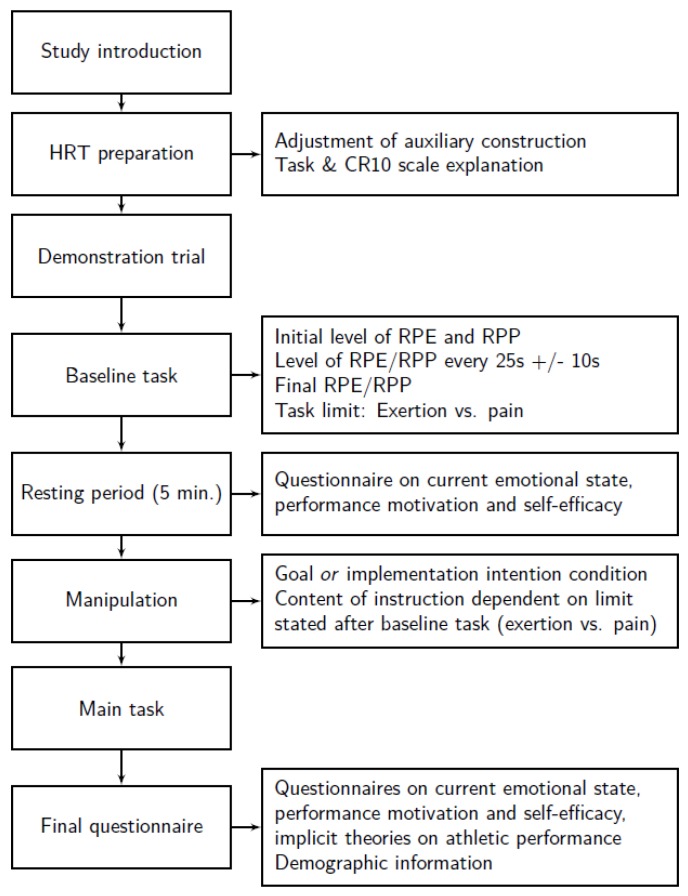
Flowchart of the study procedure. The procedure of the baseline task and the main task was analogous.

Participants were then given instruction to form a goal or an implementation intention for the upcoming main task. In both conditions, the content of the instructions depended on whether participants terminated the baseline task due to sensations of exertion or pain. In the goal condition, participants rehearsed the task *(“Even if my exertion [pain] becomes very high, the task requires to persist for as long as possible while avoiding contacts between the rings!”*). In the implementation intention condition, participants instead set a goal (i.e., “*I want to persist for as long as possible while avoiding contacts between the rings!*”) and added an if-then plan: *“And if my exertion [pain] becomes too high, then I tell myself: I can still keep going*!”. After that, participants started with the main task following the same procedure as in the baseline task. The task concluded with questionnaires regarding their current emotional state, performance motivation and self-efficacy. Finally, we measured their implicit theories on athletic performance and assessed demographic information. Figure A1 illustrates the study procedure in a flowchart.

## 3. Results

### 3.1. Data Analysis

As preliminary analyses, we compared goal and implementation intentions regarding task experience and baseline endurance performance. These comparisons were conducted with regression analyses. Correlations between the implicit theories (entity, incremental and mind-over-body) were calculated using Pearson correlations. Homogeneity of variance and normality assumption were tested using the Levene’s test and the Shapiro-Wilk test, respectively.

To address our main research question, we ran a series of regression models. We specified time-to failure and errors in the main task as dependent variables indicating endurance performance, always adjusting for the corresponding baseline values. We specified Condition (0 = goal, 1 = implementation intention) and Limit (0 = exertion, 1 = pain) as dummy predictors and beliefs (entity, incremental, mind-over-body) as continuous predictors. There were no significant differences between the group variances of time-to-failure und errors in the baseline and the main task, all *ps* ≥ 0.123. The dependent variables were not normally distributed, all *ps* < 0.001.

All analyses were run in the statistical software environment R (3.3.6, [53]). Plots were created using GGPLOT2 (3.2.1, [54]), sjPlot [55] and the graphical elements of the University of Konstanz [56]. The regression analyses were conducted with the robcov function of the rms package [57], which provides robust estimates of the standard errors. Regression tables were designed using texreg [58]. Significance is assumed when *p* < 0.05.

### 3.2. Preliminary Analyses

#### 3.2.1. Task Experience and Beliefs

There were no differences between goal and implementation intention condition regarding performance motivation, self-efficacy, task satisfaction, or positive and negative feelings, all *b*s ≤ 0.46 *p*s ≥ 0.134. More importantly, participants in both conditions displayed similar levels of incremental beliefs, *b* = −0.09, *p* = 0.529, entity beliefs, *b* = 0.005, *p* = 0.978, and mind-over-body beliefs, *b* = −0.18, *p* = 0.381. Entity and incremental beliefs were negatively associated, *r*(58) = −0.35, *p* < 0.006, as were entity and mind-over-body beliefs, *r*(58) = −0.31, *p* = 0.015. Incremental and mind-over-body beliefs were positively correlated, *r*(58) = 0.44, *p* < 0.001. We found no condition differences regarding average RPE in the baseline task, *b* = 0.34, *p* = 0.417, or in the main task, *b* = 0.19, *p* = 0.681. Analogously, there were no differences between conditions in the average RPP in the baseline task, *b* = 0.20, *p* = 0.685, or in the main task, *b* = 0.66, *p* = 0.240.

#### 3.2.2. Baseline Performance

**Figure 2:**
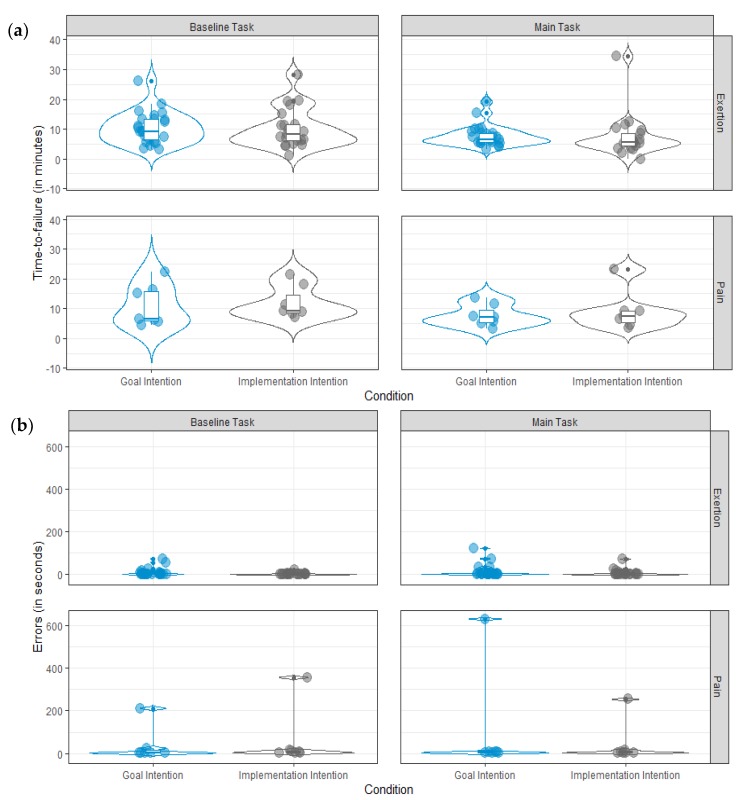
Violin plots and boxplots of (**a**) time-to-failure and (**b**) errors as a function of Condition (goal vs. implementation intention) and the reason for T1 task termination (Limit: exertion vs. pain).

There were no differences in time-to-failure (in minutes) between the goal (*M* = 10.5, *SD* = 5.5) and the implementation intention condition (*M* = 10.6, *SD* = 6.3), *b* = 0.14, *p* = 0.925. Similarly, no differences between the goal (*M* = 15.2, *SD* = 39.4) and the implementation intention condition (*M* = 16.3, *SD* = 66.9) emerged with respect to errors (in seconds), *b* = 1.03, *p* = 0.942. Figure 2 illustrates time-to-failure (Figure 2a) and errors (Figure 2b) in the baseline and the main task as a function of Condition and Limit.

In general, time-to-failure was significantly longer in the baseline task (*M* = 10.6, *SD* = 5.8) than in the main task (*M* = 7.7, *SD* = 5.2), *b* = −2.87, *p* < 0.001. Errors did not differ significantly between the baseline task (*M* = 15.7, *SD* = 53.5) and the main task (*M* = 23.8, *SD* = 88.3), *b* = 8.06, *p* = 0.274.

### 3.3. Performance in the Main Task

#### 3.3.1. Time-to-Failure

##### Conditions and Limit

Comparisons of goal versus implementation intention condition and of exertion versus pain as limits revealed no significant effects, *b* = 0.07, *p* = 0.944 (Table 2, Model 1), and *b* = 0.14, *p* = 0.921 (Table 2, Model 2), respectively, and there was no significant interaction between Condition and Limit, *b* = 0.90, *p* = 0.759 (Table 2, Model 3).

##### Entity Beliefs

**Figure 3:**
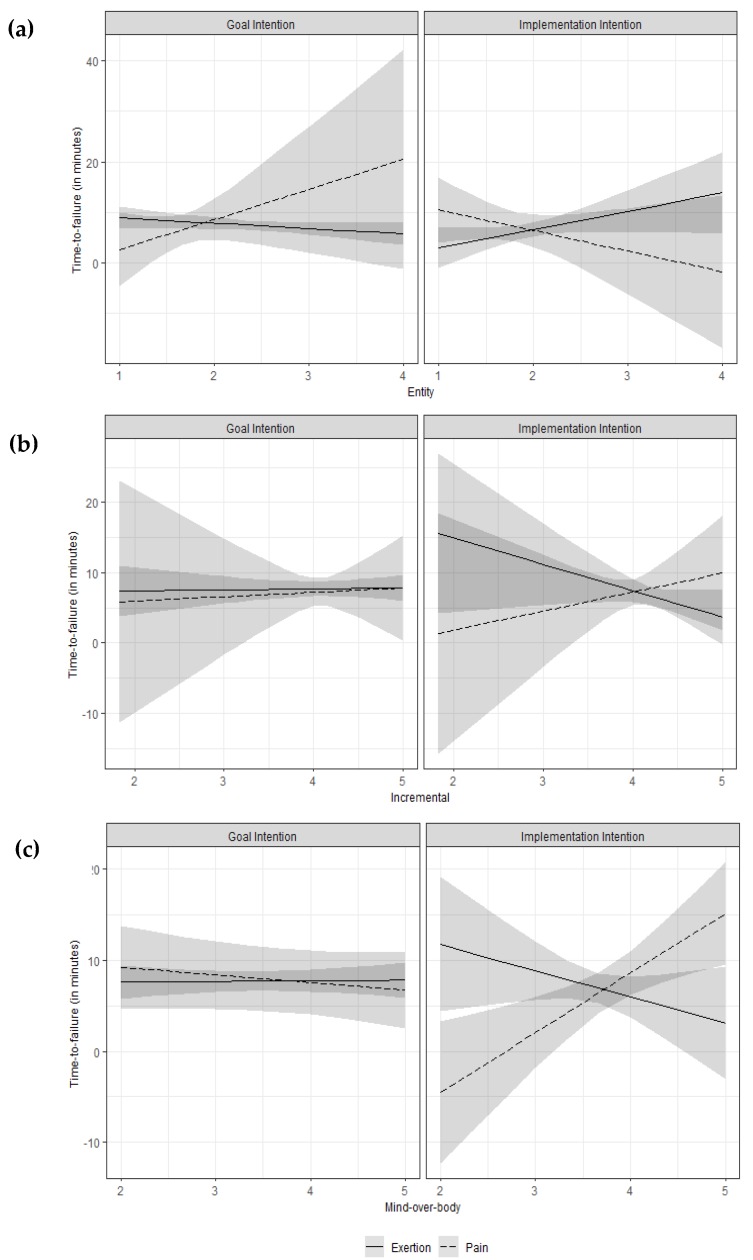
Estimated time-to-failure as a function of Condition (goal vs. if-then plan), Limit (reason for baseline task termination: exertion vs. pain) and (**a**) entity beliefs, (**b**) incremental beliefs and (**c**) mind-over-body beliefs. Higher values of entity beliefs represent a stronger belief that athletic ability is stable and fixed, while higher values of incremental beliefs represent a stronger belief that athletic ability is changeable by effort and training. Finally, higher values of mind-over-body beliefs represent a stronger belief that the mind (i.e., mental factors) rather than then the body (i.e., physical factors) limits athletic performance.

We added the entity beliefs to the variables in Model 3 and specified all possible interactions (Table 2, Model 4). This resulted in a significant effect of Condition, *b* = −10.57, *p* = 0.016, that was governed by significant two-way interactions with Limit, *b* = 28.47, *p* = 0.013, and entity beliefs, *b* = 4.67, *p* = 0.026, and by a significant three-way interaction with Limit and entity beliefs, *b* = −14.69, *p* = 0.020. To ease the interpretation this three-way interaction, we plotted time-to-failure as a function of Condition, Limit, and the entity beliefs in Figure 3a. In the goal condition, stronger entity beliefs were associated with decreasing time-to failure when participants felt limited by feelings of exertion but with increasing time-to-failure when they felt limited by feelings of pain. This was reversed in the implementation intention condition, where stronger entity beliefs were associated with increasing time-to-failure when participants felt limited by feelings of exertion but with decreasing time-to-failure when they felt limited by feelings of pain.

##### Incremental Beliefs

In an analogous fashion, we added the incremental beliefs to the variables in Model 3 and specified all possible interactions (Table 2, Model 5). This revealed no significant effects, *p*s ≥ 0.112. However, as can be seen in Figure 3b, the association between Limit and incremental beliefs especially in the implementation intention condition was reversed in comparison to the analysis of entity beliefs.

##### Mind-over-body Beliefs

Next, we added mind-over-body beliefs in the same fashion (Table 2, Model 6). This revealed a significant interaction between Condition and Limit, *b* = −38.45, *p* = 0.003, that was governed by a three-way interaction of Condition, Limit and mind-over-body beliefs, *b* = 10.32, *p* = 0.002 (see Figure 3c). While time-to-failure was not affected by limits and mind-over-body beliefs in the goal condition, stronger mind-over-body beliefs in the implementation intention condition were associated with decreasing time-to-failure when participants felt limited by feelings of exertion but with increasing time-to-failure when they felt limited by feelings of pain.

##### Joint Analysis of Beliefs

In a final step, we simultaneously added entity, incremental, and mind-over-body beliefs to examine which beliefs are most important for time-to-failure when controlling for the influence of other beliefs (Table 2, Model 7). We found that the three-way interaction effect involving entity became nonsignificant, *b* = −9.01, *p* = 0.069, while the three-way interaction effect involving mind-over-body beliefs remained significant, *b* = 9.20, *p* = 0.006. The interaction effect involving incremental beliefs remained nonsignificant, *b* = −0.26, *p* = 0.962. This pattern of results suggests that mind-over-body beliefs in concert with perceived limits of performance (exertion vs. pain) were an important determinant of implementation intention effects on time-to-failure.

#### 3.3.2. Errors

##### Condition and Limit

Comparisons of goal versus implementation intention condition and of exertion versus pain as limiting factors regarding errors revealed no significant effects, *b* = −16.19, *p* = 0.244, and *b* = 7.22, *p* = 0.530, respectively, and there was no significant interaction between Condition and Limit, *b* = −83.49, *p* = 0.169 (Table A1, Models 1–3).

##### Moderation by Entity, Incremental and Mind-over-body Beliefs

**Figure A2:**
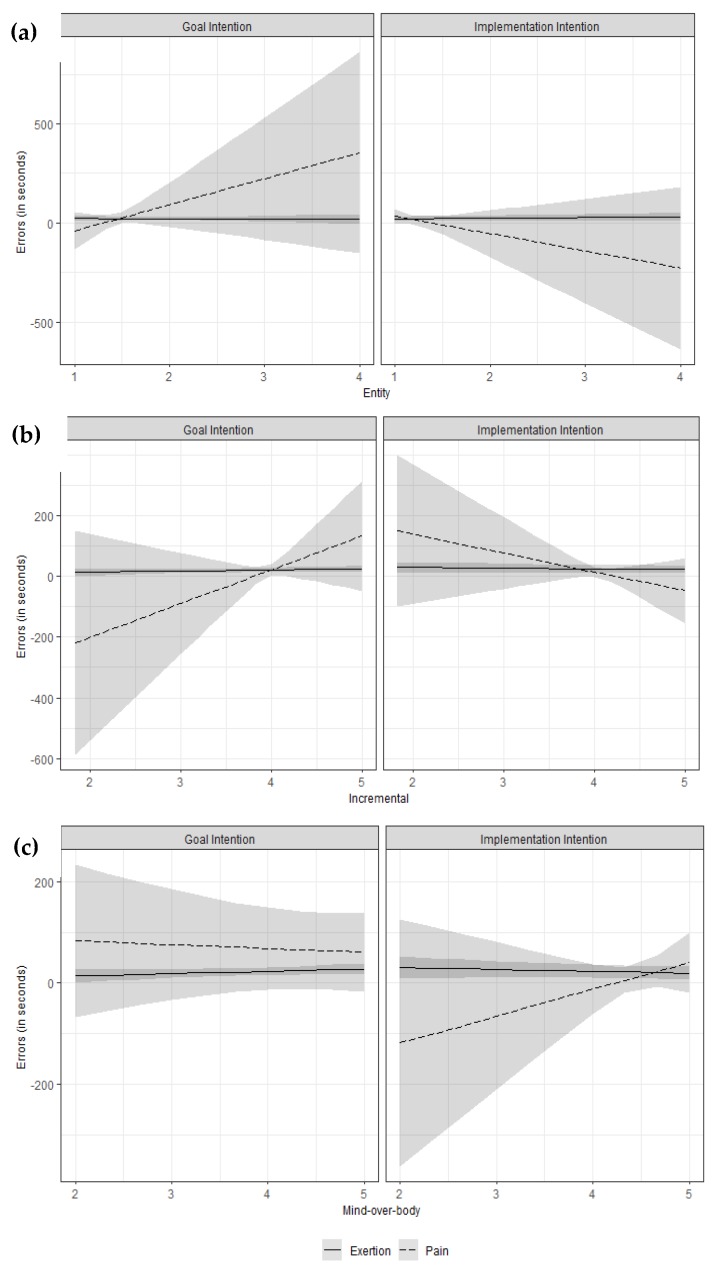
Estimated errors as a function of Condition (goal vs. if-then plan), Limit (reason for baseline task termination: exertion vs. pain) and (**a**) entity beliefs, (**b**) incremental beliefs and (**c**) mind-over-body beliefs. Higher values of entity beliefs represent a stronger belief that athletic ability is stable and fixed, while higher values of incremental beliefs represent a stronger belief that athletic ability is changeable by effort and training. Finally, higher values of mind-over-body beliefs represent a stronger belief that the mind (i.e., mental factors) rather than then the body (i.e., physical factors) limits athletic performance.

Analogous to the analysis of time-to-failure, we added the beliefs and specified interactions with all variables in Model 3 (Table A1, Models 4–6). These analyses did not yield any significant effects, *p*s ≥ 0.073. Errors are plotted as a function of Condition, Limit, and the respective beliefs in Figure A2.

##### Joint Analysis of Beliefs

In a final step, we simultaneously added entity, incremental, and mind-over-body beliefs and their interactions with Condition and Limit, which revealed no significant effects, *p*s ≥ 0.055 (Table A1, Model 7).

## 4. Discussion

The aim of this study was to advance research on the effects of forming implementation intentions on muscular endurance performance. Specifically, we investigated the effectiveness of implementation intentions tailored to experienced limits of endurance performance (i.e., perceptions of exertion versus pain) conjointly with potentially moderating effects of individual differences in implicit theories about the stability versus malleability of athletic performance (i.e., entity versus incremental beliefs) and about the limitation of athletic performance by mental versus physical factors (i.e., mind-over-body beliefs).

In line with prior research [18,31], forming goal versus implementation intentions did not improve endurance performance in terms of time-to-failure or errors, even though the if-then plans were tailored to previously experienced limits of endurance performance. However, individual differences in implicit theories emerged as a moderating variable: In the implementation intention condition, stronger entity beliefs were associated with increasing time-to-failure when participants planned to ignore exertion but with decreasing time-to-failure when they planned to ignore pain. In contrast, stronger entity beliefs in the goal condition were associated with decreasing time-to-failure when the goal pertained to exertion but with increasing time-to-failure when the goal pertained to pain. Interestingly, this pattern of results was reversed with regard to incremental beliefs (although this pattern itself was not significant) and even more so with regard to mind-over-body beliefs. This observation makes sense when considering that entity beliefs were negatively correlated with incremental and mind-over-body beliefs, which in turn were positively correlated with each other.

The role of mind-over-body beliefs seems particularly intriguing because beliefs about the nature of limits to athletic performance have so far garnered little or no scientific attention. Our findings might be tentatively interpreted as follows: Participants who believe that mental factors ultimately limit athletic performance (i.e., strong mind-over-body beliefs) and who had just terminated the baseline task because of such a mental factor (i.e., perceived exertion) likely interpret their exertion as signal that their personal limit is reached. This might undermine the effectiveness of implementation intentions, especially when the plan requires them to ignore this signal. The very same plan might, however, be viable for participants who likewise had just terminated the baseline task because of exertion but do not believe in the mind’s role in limiting performance (i.e., weak mind-over-body beliefs). These participants are unlikely to interpret exertion as signaling the reaching of a limit, leaving room for implementation intention effects to unfold. By analogy, plans targeting the perception of pain might be more viable for participants who terminated the baseline task because of pain (i.e., a bodily factor) and hold strong rather than weak mind-over-body beliefs. It seems promising for future research to follow this line of reasoning up by using implementation intentions targeting perceptions that were not the reason for previous task termination (e.g., planning to ignore pain after terminating the baseline task due to exertion).

Of course, our reasoning rests on the assumption that exertion and pain are two distinguishable perceptions that people conceive of as representing mental versus bodily factors, respectively. This view is supported by the literature, which describes perceived exertion as an indicator of mental strain [59] and perceived pain as corresponding to bodily processes [33]. Accordingly, our results suggest that the contents of implementation intentions should be aligned with people’s implicit beliefs about the (reaching of) limits of athletic abilities in order to work effectively. In our study, this was particularly important with regard to entity and mind-over-body beliefs.

In contrast to our initial assumptions, we did not observe a direct impact of entity and incremental beliefs on implementation intention effects. As we have argued in the introduction, it was plausible to assume that entity and incremental beliefs about athletic ability have direct (although potentially different) consequences for implementation intentions. However, we found no significant effects of incremental beliefs at all and no direct effects of entity beliefs. Although, the observation that stronger entity beliefs were associated with longer time-to-failure when the implementation intention targeted exertion can be explained with the existing literature on implicit theories: from an entity perspective, exertion signals a deficit because people with talent do not need to invest effort in order to achieve something [35,60]. Therefore, being confronted with sensations of exertion in the baseline task, they probably were more likely to respond well to an implementation intention focused on ignoring exertion. This reasoning can be applied vice versa to an incremental perspective: here, exertion is an unavoidable part of development and necessary for transforming skills into accomplishments [35,61]. Accordingly, participants holding stronger incremental beliefs would presumably be wary using an implementation intention targeting the ignorance of exertion, an allegation that is implied by the visualization of time-to-failure in Figure 3b. However, in our study we assessed implicit theories after the completion of the baseline and the main task to avoid undesired effects of the measurement on performance. Therefore, future research should complement our approach with a design in which implicit theories are assessed prior to the endurance task.

Our study has important implications for the design of implementation intentions in studies investigating athletic performance. It provides guidance on how implementation intentions should be constructed in order to overcome exercise-related obstacles. In former studies, all participants formed the same implementation intention targeting a single obstacle known to be of general relevance (i.e., perceptions of pain or effort; e.g., [18]). This has yielded mixed results, however, and our findings suggest an intricate interplay between beliefs about athletic performance and the (reaching of) limits as possible reason for this inconsistency. It thus seems advisable to tailor implementation intentions specifically to people’s beliefs about performance and its limits, for instance, by avoiding that the implementation intentions requiring to ignore a factor that they interpret as signal that their personal (mental or physical, as the case may be) limits are reached. All in all, the results further the assumption that a generalizable application of if-then plans in exercise contexts is not always effective and provides an explanation as to why similar studies did not observe positive effects for plans [18,31]. Instead, an individual consideration of exercisers’ implicit beliefs in what limits their performance is necessary in order to improve muscular endurance performance. This study therefore makes a strong contribution for the research on the use of if-then plans in (muscular) endurance performance by specifying how to tailor implementation intentions to exercisers’ needs.

As mentioned above, further studies should provide participants with implementation intentions after having assessed their implicit theories. This would allow, for instance, to rule out the possibility that participants’ expressed believes (i.e., entity, incremental, and mind-over-body) were affected by their earlier experiences of exertion or pain during the task, and/or by the effectiveness of the corresponding if-then plans. Until such data is available, causal interpretations must remain tentative. Another intriguing question pertains to the effects of implementation intentions tailored to perceptions that participants did not attribute to task termination. That is, participants who terminate the baseline task because of exertion would plan to ignore their pain, whereas participants who terminate the baseline task because of pain would plan to ignore their exertion. Such an approach avoids mismatches between the content of implementation intentions and participants’ beliefs, as the plan could no longer target a perception that is seen as signaling the reaching of a limit. Finally, it seems promising to experimentally manipulate participants’ believes about athletic ability rather than measuring it (e.g., [60,62,63]). This would permit stronger causal inferences and therefore provide additional insights in the interplay of implementation intentions and implicit theories.

That said, our findings already contribute to literature on the moderators of implementation intention effects [64]. This literature has mainly focused on individual difference variables like social anxiety [65], conscientiousness [66], and perfectionism [67], but also on situational moderators like mindsets [68]. Here, we combined both approaches by demonstrating an interaction of participants’ general beliefs about athletic ability as a trait (i.e., entity, incremental, mind-over-body) and the limiting factors of their performance as a state (i.e., perceptions of exertion and pain). As such, our research reinforces calls for investigating moderators of implementation intention effects and highlights the importance of considering both traits and states in doing so. This might be particularly relevant with respect to physical exercise and endurance performance, given the currently conflicting sets of findings in this domain.

Finally, and reaching beyond theoretical considerations, our study has implications for designing interventions that help recreational athletes to initiate and maintain regular muscular exercise. Despite common knowledge that physical activity is important for health [1,2], half of the people who want to become (and stay) physically active fail to do so [69]. In the domain of muscular endurance, an important cause for such failures might be that the training is perceived as effortful and is accompanied by uncomfortable sensations like pain [70]. Given their effectiveness across domains, it is therefore not surprising that implementation intentions are frequently recommended as a self-regulation strategy in the sports context [29,70,71,72,73] and tailored primarily to the regulation of effort and pain when it comes to improving muscular endurance [18,30,31]. However, our results indicate that conveying implementation intentions to recreational athletes is not without its hooks. For instance, it might seem intuitive to advice recreational athletes to ignore their perceptions of effort when they perceive an exercise as too effortful by means of forming an implementation intention. Our data suggest, however, that this might backfire if these athletes interpret their effort as signaling that an ultimate performance limit has been reached. Against this background and considering the conflicting findings regarding implementation intention effects in the domain of endurance performance, further research seems necessary to develop implementation intentions that effectively enhance muscular endurance in applied settings. As a first and important step, our study showed how individual differences in believes and perceived performance limits could be used to design implementation intentions.

One could argue that the results of this study are not easily applicable to the broad population of frequently exercising recreational athletes, as the study sample consists of students with reasonable variation in regard to exercise motivation and athletic experience. It is possible that the influence of implicit theories on athletic ability and of beliefs on the limitations of athletic performance is different in populations of more experienced exercisers, as they might already have experienced the boundaries of their bodily resources and thus possess an enhanced body awareness. These experiences might have an effect on self-regulation strategies such as implementation intentions. Future research should extend our results by investigating the impact of individual beliefs about athletic ability and of perceived limits of endurance performance particularly in hobby, amateur, and professional endurance athletes.

Moreover, our results concerning the influence of mind-over-body beliefs should be taken with a grain of salt. On the one hand, we think that these beliefs are important for evaluating the effectiveness of psychological interventions in the exercise domain, and this impression is reinforced by our observation that mind-over-body beliefs played an even more powerful role than entity and incremental beliefs. On the other hand, our findings rest on an ad hoc constructed scale in accordance with and complementing the established CNAAQ-2. The rather weak internal consistency of the three constituent items indicates that we tapped into a rather complex construct that might be difficult to capture in full with only three items. Given the relevance of these items in explaining the effectiveness of if-then plans according to our data, it might be worthwhile to develop and psychometrically test a dedicated scale for assessing mind-over-body beliefs.

## 5. Conclusions

In this study, we investigated the effects of if-then planning on performance in a static muscular endurance task as well as potential moderators of these effects. Our findings point to an important role of individual beliefs about athletic ability (i.e., entity, incremental, and mind-over-body) as well as the perceived limits of endurance performance (i.e., exertion and pain). The content of an implementation intention needs to be congruent with these beliefs and limits in order to improve performance: if, for instance, people plan to ignore a perception (e.g., exertion) that is interpreted as signaling the reaching of a limit according to their beliefs about athletic ability (e.g., mind-over-body), performance seems to deteriorate. In contrast, planning to ignore a perception that is not interpreted as signaling the reaching of a limit seems to promote performance. The findings of this study increase the current knowledge on moderators of if-then planning and add to the existing research on the efficacy of implementation intentions on exercise performance. We hasten to add that our interpretations remain tentative until further experimental evidence is available that systematically investigates the interplay of plan contents, beliefs, and perceptions. Nevertheless, our data provide solid grounds for suggesting that implementation intentions should be carefully tailored to what recreational athletes believe about athletic performance and their own limits. Such tailoring of implementation intentions might be a powerful tool to assist exercisers with staying on track even during effortful muscular endurance tasks and deal with aching muscles, ultimately helping them to enhance their physical fitness and health. Therefore, this study provides a valuable contribution to research on health-related behavior by providing insights on how to optimize the application of well-researched psychological strategies in the exercise context. Finally, it is important to note that in the health setting, sports and exercise is often performed at low intensities that do not push exercisers to their effort- and pain-related limits. Therefore, low intensity exercise is likely to pose different self-regulatory challenges that threaten long-term exercise adherence: For many people, exercise might simply be boring. Recently, it has been proposed that boredom acts as a powerful motivator for seeking out more rewarding behavioral alternatives [74], and these might be at odds with one’s goal of becoming fit and healthy. Thus, self-regulatory control is required to keep going despite being bored [74]. Therefore, future research should investigate how if-then plans can be tailored to the challenges of low intensity exercise, to help aspiring exercisers deal with these self-regulatory challenges.

## Figures and Tables

**Table 1:** Items used for measuring entity and incremental beliefs (CNAAQ-2; [42]), complemented by three items measuring implicit theories about the body versus the mind as limiting factors of athletic performance (i.e., mind-over-body beliefs).

First-Order Variable	Item
Entity	1. You have a certain level of ability in sport and you cannot really do much to change that level.
	2. Even if you try, the level you reach in sport will change very little.
	3. It is difficult to change how good you are at sport.
	4. You need to have certain ‘gifts’ to be good at sport.
	5. To be good at sport, you need to be born with the basic qualities which allow you success.
	6. To be good at sport you need to be naturally gifted.
Incremental	7. To be successful in sport you need to learn techniques and skills and practice them regularly.
	8. You need to learn and to work hard to be good at sport.
	9. To reach a high level of performance in sport, you must go through periods of learning and training.
	10. In sport, if you work hard at it, you will always get better.
	11. How good you are at sport will always improve if you work at it.
	12. If you put enough effort into it, you will always get better at sport.
Mind-over-body	13. The body sets limits to athletic performance that cannot be overcome.
	14. Mental attitude does not play a role in sports, if the physical preconditions are not met.
	15. One can always enhance one’s athletic performance through mental processes.

**Table 2:** Linear regression models for explaining time-to-failure in the main task of the HRT. The intercept represents the expected time-to-failure in the main task when all other predictors equal 0. Numbers in parentheses are robust standard errors. Baseline: Condition = Goal Intention, Limit (reason for baseline task termination) = Exertion.

Variable	Model 1	Model 2	Model 3	Model 4	Model 5	Model 6	Model 7
Intercept	1.59	1.60	1.68	4.59	2.01	1.97	5.42
	(1.81)	(1.52)	(1.88)	(2.03) *	(3.44)	(2.26)	(3.86)
Time-to-failure Baseline Task	0.57 ***	0.57 ***	0.57 ***	0.51 ***	0.48 ***	0.52 ***	0.45 ***
	(0.17)	(0.17)	(0.17)	(0.11)	(0.10)	(0.12)	(0.08)
Condition = II	0.07		−0.14	−10.57 *	15.33	9.97	4.54
	(1.03)		(1.11)	(4.23)	(10.34)	(8.03)	(7.67)
Limit = Pain		0.14	−0.30	−13.30	−2.33	3.37	−20.44
		(1.44)	(1.94)	(8.30)	(15.84)	(4.15)	(14.66)
II × Pain			0.90	28.47 *	−23.67	−38.45 **	−14.97
			(2.94)	(11.16)	(24.33)	(12.18)	(19.49)
Entity				−1.06 (0.65)			−1.08 ° (0.64)
II × Entity				4.67 * (2.04)			3.43 ** (1.21)
Pain × Entity				7.00 (4.71)			7.04 ° (4.13)
II × Pain × Entity				−14.69 * (6.12)			−9.01 ° (4.84)
Incremental					0.15 (0.78)		−0.00 (0.78)
II × Incremental					3.89 (2.46)		−2.15 (1.91)
Pain × Incremental					0.46 (3.89)		2.89 (4.87)
II × Pain × Incremental					5.98 (5.99)		−0.26 (5.46)
Mind-over-body						0.06 (0.51)	−0.04 (0.54)
II × Mind-over-body						−2.93 (2.20)	−1.09 (1.42)
Pain × Mind-over-body						−0.89 (1.03)	−1.38 (1.84)
II × Pain × Mind-over-body						10.32 ** (3.23)	9.20 ** (3.19)
Num. obs.	60	60	60	60	60	60	60
R2	0.42	0.42	0.42	0.54	0.50	0.54	0.64
Adj. R2	0.40	0.40	0.38	0.47	0.42	0.47	0.50
L.R.	32.38	32.39	32.54	47.04	41.10	46.48	60.92

*** p < 0.001, ** *p* < 0.01, * *p* < 0.05, ° *p* < 0.1; L.R., likelihood ratio; Num. obs., number of observations; Adj. R2, adjusted R2; II = Implementation Intention.

## Appendix A

**Table A1:** Linear regression model for explaining errors in the main task in the HRT. The intercept represents the expected mean value of errors in the main task when all other predictors are equal to 0. Numbers in parentheses are robust standard errors. Baseline: Condition = Goal Intention, Limit (reason for baseline task termination) = Exertion.

Variable	Model 1	Model 2	Model 3	Model 4	Model 5	Model 6	Model 7	
Intercept	11.08	2.14	0.42	1.70	−15.13	−16.97 °	−18.94	
	(8.19)	(4.26)	(5.39)	(17.60)	(10.64)	(9.57)	(17.64)	
Condition = II	−16.19		3.19	−6.23	27.14 °	32.76 °	24.12	
	(13.76)		(5.29)	(16.10)	(14.89)	(17.90)	(20.04)	
Errors Baseline Task	1.29 *	1.27 *	1.31*	1.38 **	1.28 **	1.38 **	1.26 ***	
	(0.57)	(0.57)	(0.51)	(0.45)	(0.44)	(0.51)	(0.31)	
Limit = Pain		7.22	47.25	−194.70	−426.78	93.25	−818.63	
		(11.42)	(40.60)	(143.41)	(341.01)	(119.08)	(502.54)	
II × Pain			−83.491	297.44	657.46	−355.29	1017.63 °	
			(60.025)	(185.30)	(475.46)	(280.37)	(569.84)	
Entity				−0.90 (6.07)			−1.00 (4.54)	
					
II × Entity				4.42 (6.18)			3.58 (4.84)	
					
Pain × Entity				132.57 (98.07)			140.07 (88.52)	
					
II × Pain × Entity				−223.02 (137.78)			−188.58 ° (101.99)	
					
Incremental					3.85 (2.34)		1.36 (2.33)	
					
II × Incremental					−5.94 ° (3.38)		−0.87 (4.49)	
					
Pain × Incremental					106.96 (85.97)		217.79 ° (128.09)	
					
II × Pain × Incremental					−167.02 (119.70)		−285.29 * (145.04)	
					
Mind-over-body						4.56 (3.25)	4.49 (3.21)	
					
II × Mind-over-body						−8.16 °	−7.16	
						(4.72)	(5.42)	
Pain × Mind-over-body						−12.18	−86.27	
						(25.06)	(55.84)
II × Pain × Mind-over-body						68.80	122.40 °	
						(58.14)	(72.59)	
Num. obs.	60	60	60	60	60	60	60	
R2	0.62	0.61	0.66	0.72	0.70	0.68	0.80	
Adj. R2	0.60	0.60	0.63	0.68	0.65	0.62	0.73	
L.R.	57.68	56.52	64.55	77.00	72.22	67.57	97.69	

*** *p* < 0.001, ** *p* < 0.01, * *p* < 0.05, ° *p* < 0.1; L.R., likelihood ratio; Num. obs., number of observations; Adj. R2, adjusted R2; II Implementation Intention.

## References

[1] Ruiz J.R., Castro-Piñero J., Artero E.G., Ortega F.B., Sjöström M., Suni J., Castillo M.J. (2009). Predictive validity of health-related fitness in youth: A systematic review. Br. J. Sports Med..

[2] Ortega F.B., Ruiz J.R., Castillo M.J., Sjöström M. (2008). Physical fitness in childhood and adolescence: A powerful marker of health. Int. J. Obesity.

[3] Smith J.J., Eather N., Morgan P.J., Plotnikoff R.C., Faigenbaum A.D., Lubans D.R. (2014). The health benefits of muscular fitness for children and adolescents: A systematic review and meta-analysis. Sports Med..

[4] Ruiz J.R., Sui X., Lobelo F., Lee D.-C., Morrow J.R., Jackson A.W., Hébert J.R., Matthews C.E., Sjöström M., Blair S.N. (2009). Muscular strength and adiposity as predictors of adulthood cancer mortality in men. Cancer Epidemiol. Biomark. Prev..

[5] García-Hermoso A., Cavero-Redondo I., Ramírez-Vélez R., Ruiz J.R., Ortega F.B., Lee D.-C., Martínez-Vizcaíno V. (2018). Muscular strength as a predictor of all-cause mortality in an apparently healthy population: A systematic review and meta-analysis of data from approximately 2 million men and women. Arch. Phys. Med. Rehabilit..

[6] Artero E.G., Lee D.-C., Lavie C.J., España-Romero V., Sui X., Church T.S., Blair S.N. (2012). Effects of muscular strength on cardiovascular risk factors and prognosis. J. Cardiopulm. Rehabilit. Prev..

[7] Ortega F.B., Artero E.G., Jiménez-Pavón D., Ruiz J.R. (2018). Role of physical activity and fitness in the promotion of metabolic and overall health. Eur. J. Hum. Mov..

[8] Piercy K.L., Troiano R.P., Ballard R.M., Carlson S.A., Fulton J.E., Galuska D.A., George S.M., Olson R.D. (2018). The physical activity guidelines for Americans. JAMA.

[9] Ozemek C., Lavie C.J., Rognmo Ø. (2019). Global physical activity levels—Need for intervention. Prog. Cardiovasc. Dis..

[10] Allen D.G., Lamb G.D., Westerblad H. (2008). Skeletal muscle fatigue: Cellular mechanisms. Physiol. Rev..

[11] Amann M., Calbet J.A.L. (2008). Convective oxygen transport and fatigue. J. Appl. Physiol..

[12] Burnley M., Jones A.M. (2007). Oxygen uptake kinetics as a determinant of sports performance. Eur. J. Sport Sci..

[13] Secher N.H., Seifert T., Van Lieshout J.J. (2008). Cerebral blood flow and metabolism during exercise: Implications for fatigue. J. Appl. Physiol..

[14] Marcora S.M., Staiano W. (2010). The limit to exercise tolerance in humans: Mind over muscle?. Eur. J. Appl. Physiol..

[15] Marcora S.M., Staiano W., Manning V. (2009). Mental fatigue impairs physical performance in humans. J. Appl. Physiol..

[16] Marcora S.M. (2008). Do we really need a central governor to explain brain regulation of exercise performance?. Eur. J. Appl. Physiol..

[17] Marcora S.M., Bosio A., de Morree H.M. (2008). Locomotor muscle fatigue increases cardiorespiratory responses and reduces performance during intense cycling exercise independently from metabolic stress. Am. J. Physiol. Regul. Integr. Comp. Physiol..

[18] Wolff W., Bieleke M., Hirsch A., Wienbruch C., Gollwitzer P.M., Schüler J. (2018). Increase in prefrontal cortex oxygenation during static muscular endurance performance is modulated by self-regulation strategies. Sci. Rep..

[19] Gollwitzer P.M., Sheeran P. (2006). Implementation intentions and goal achievement: A meta-analysis of effects and processes. Adv. Exp. Soc. Psychol..

[20] Martiny-Huenger T., Bieleke M., Oettingen G., Gollwitzer P.M., Deutsch R., Gawronski B., Hofmann W. (2017). From thought to automatic action: Strategic and incidental action control by if-then planning. Reflective and Impulsive Determinants of Human Behavior.

[21] Bélanger-Gravel A., Godin G., Amireault S. (2013). A meta-analytic review of the effect of implementation intentions on physical activity. Health Psychol. Rev..

[22] Adriaanse M.A., Vinkers C.D.W., de Ridder D.T.D., Hox J.J., de Wit J.B.F. (2011). Do implementation intentions help to eat a healthy diet? A systematic review and meta-analysis of the empirical evidence. Appetite.

[23] Orbell S., Sheeran P. (2000). Motivational and volitional processes in action initiation: A field study of the role of implementation intentions. J. Appl. Soc. Psychol..

[24] Gollwitzer P.M. (2014). Weakness of the will: Is a quick fix possible?. Motiv. Emot..

[25] Gollwitzer P.M. (1999). Implementation intentions: Strong effects of simple plans. Am. Psychol..

[26] Janczyk M., Dambacher M., Bieleke M., Gollwitzer P.M. (2015). The benefit of no choice: Goal-directed plans enhance perceptual processing. Psychol. Res..

[27] Triandis H.C. (1977). Interpersonal Behavior.

[28] Hagger M.S., Montasem A. (2009). Implementing intentions to drink a carbohydrate-electrolyte solution during exercise. J. Sports Sci..

[29] Achtziger A., Gollwitzer P.M., Sheeran P. (2008). Implementation intentions and shielding goal striving from unwanted thoughts and feelings. Pers. Soc. Psychol. Bull..

[30] Thürmer J.L., Wieber F., Gollwitzer P.M. (2017). Planning and performance in small groups: Collective implementation intentions enhance group goal striving. Front. Psychol..

[31] Bieleke M., Wolff W. (2017). That escalated quickly—Planning to ignore RPE can backfire. Front. Physiol..

[32] Marcora S.M., Goldstein E.B. (2010). Effort: Perception of. Encyclopedia of Perception.

[33] Pageaux B. (2016). Perception of effort in exercise science: Definition, measurement and perspectives. Eur. J. Sport Sci..

[34] Dweck C.S. (1999). Self-Theories. Their Role in Motivation, Personality, and Development.

[35] Dweck C.S., Leggett E.L. (1988). A social-cognitive approach to motivation and personality. Psychol. Rev..

[36] Dweck C.S., Gollwitzer P., Bargh J.A. (1996). Implicit theories as organizers of goals and behavior. The Psychology of Action: Linking Cognition and Motivation to Behavior.

[37] Dweck C.S., Chiu C.-Y., Hong Y.-Y. (1995). Implicit theories and their role in judgments and reactions: A word from two perspectives. Psychol. Inq..

[38] Ross M. (1989). Relation of implicit theories to the construction of personal histories. Psychol. Rev..

[39] Job V., Walton G.M., Bernecker K., Dweck C.S. (2013). Beliefs about willpower determine the impact of glucose on self-control. Proc. Natl. Acad. Sci. USA.

[40] Napolitano C.M., Job V. (2018). Assessing the implicit theory of willpower for Strenuous Mental Activities Scale: Multigroup, across-gender, and cross-cultural measurement invariance and convergent and divergent validity. Psychol. Assess..

[41] Li W., Lee A. (2004). A review of conceptions of ability and related motivational constructs in achievement motivation. Quest.

[42] Biddle S.J.H., Wang C.K.J., Chatzisarantis N.L.D., Spray C.M. (2003). Motivation for physical activity in young people: Entity and incremental beliefs about athletic ability. J. Sports Sci..

[43] Spray C.M., Wang C.J., Biddle S.J.H., Chatzisarantis N.L.D., Warburton V.E. (2006). An experimental test of self-theories of ability in youth sport. Psychol. Sport Exerc..

[44] Wang C.K.J., Biddle S.J.H. (2003). Intrinsic motivation towards sports in Singaporean students: The role of sport ability beliefs. J. Health Psychol..

[45] Burnette J.L., O’Boyle E.H., VanEpps E.M., Pollack J.M., Finkel E.J. (2013). Mind-sets matter: A meta-analytic review of implicit theories and self-regulation. Psychol. Bull..

[46] Burnette J.L. (2010). Implicit theories of body weight: Entity beliefs can weigh you down. Pers. Soc. Psychol. Bull..

[47] Kasimatis M., Miller M., Marcussen L. (1996). The effects of implicit theories on exercise motivation. J. Res. Pers..

[48] Gardner L.A., Vella S.A., Magee C.A. (2017). Continued participation in youth sports: The role of achievement motivation. J. Appl. Sport Psychol..

[49] Schüler J., Wolff W., Dettmers C. (2019). Exercise in multiple sclerosis: Knowing is not enough—The crucial role of intention formation and intention realization. Neurol. Ther..

[50] Giboin L.-S., Gruber M., Schüler J., Wolff W. (2019). Investigating performance in a strenuous physical task from the perspective of self-control. Brain Sci..

[51] Borg G. (1998). Borg’s Perceived Exertion and Pain Scales.

[52] Borg G. (2004). The Borg CR10 Scale Folder.

[53] R Core Team (2018). R: A Language and Environment for Statistical Computing.

[54] Wickham H. (2009). ggplot2.

[55] Lüdecke D. (2019). sjPlot: Data Visualization for Statistics in Social Science.

[56] Neth H., Gradwohl N. (2019). unikn: Graphical elements of the University of Konstanz’s corporate design.

[57] Harrell F.E. (2015). Regression Modeling Strategies. With Applications to Linear Models, Logistic and Ordinal Regression, and Survival Analysis.

[58] Leifeld P. (2013). texreg: Conversion of statistical model output in R to HTML tables. J. Stat. Softw..

[59] Borg G. (1982). Psychophysical bases of perceived exertion. Med. Sci. Sports Exerc..

[60] Hong Y.-Y., Chiu C.-Y., Dweck C.S., Lin D.M.-S., Wan W. (1999). Implicit theories, attributions, and coping: A meaning system approach. J. Pers. Soc. Psychol..

[61] Blackwell L.S., Trzesniewski K.H., Dweck C.S. (2007). Implicit theories of intelligence predict achievement across an adolescent transition: A longitudinal study and an intervention. Child Dev..

[62] Aronson J., Fried C.B., Good C. (2002). Reducing the effects of stereotype threat on African American college students by shaping theories of intelligence. J. Exp. Soc. Psychol..

[63] Chiu C.-Y., Hong Y.-Y., Dweck C.S. (1997). Lay dispositionism and implicit theories of personality. J. Pers. Soc. Psychol..

[64] Prestwich A., Kellar I. (2014). How can the impact of implementation intentions as a behaviour change intervention be improved?. Eur. Rev. Appl. Psychol..

[65] Webb T.L., Ononaiye M.S.P., Sheeran P., Reidy J.G., Lavda A. (2010). Using implementation intentions to overcome the effects of social anxiety on attention and appraisals of performance. Pers. Soc. Psychol. Bull..

[66] Webb T.L., Christian J., Armitage C.J. (2007). Helping students turn up for class: Does personality moderate the effectiveness of an implementation intention intervention?. Learn. Individ. Differ..

[67] Powers T.A., Koestner R., Topciu R.A. (2005). Implementation intentions, perfectionism, and goal progress: Perhaps the road to hell is paved with good intentions. Pers. Soc. Psychol. Bull..

[68] Wieber F., Sezer L.A., Gollwitzer P.M. (2014). Asking “why” helps action control by goals but not plans. Motiv. Emot..

[69] Rhodes R.E., de Bruijn G.-J. (2013). How big is the physical activity intention-behaviour gap? A meta-analysis using the action control framework. Br. J. Health Psychol..

[70] McCormick A., Meijen C., Marcora S.M. (2016). Psychological demands experienced by recreational endurance athletes. Int. J. Sport Exerc. Psychol..

[71] Calder A. (2009). Fatigue is no foe with recovery strategies. Sports Official Newsletter.

[72] Gregoire C. Why Runners ‘Hit the Wall,’ and What to Do about It. https://www.huffpost.com/entry/runners-psychological-mental-obstacles_n_58484917e4b08c82e8893393.

[73] Brick N.E., MacIntyre T.E., Campbell M.J. (2016). Thinking and action: A cognitive perspective on self-regulation during endurance performance. Front. Physiol..

[74] Wolff W., Martarelli C. (2020). Bored into depletion? Towards a tentative integration of perceived self-control exertion and boredom as guiding signals for goal-directed behavior. Perspect. Psychol. Sci..

